# Viability of Booby Offspring is Maximized by Having One Young Parent and One Old Parent

**DOI:** 10.1371/journal.pone.0133213

**Published:** 2015-07-29

**Authors:** Hugh Drummond, Cristina Rodríguez

**Affiliations:** Departamento de Ecología Evolutiva, Instituto de Ecología, Universidad Nacional Autónoma de México, Mexico City, Mexico; Institute of Biology, University Leipzig, GERMANY

## Abstract

It is widely expected that the quality of offspring will vary with the age of their parents and that this variation should influence animals’ choice of mates. However, theoretical predictions for age effects are contradictory and, to our knowledge, we do not know for any wild animal how the quality of offspring is affected by both parents’ ages across their lifespans, or whether mothers’ and fathers’ ages interact. We tackled this question using long-term data on a highly philopatric, insular population of the blue-footed booby (*Sula nebouxii*). In this species extra-pair paternity is most common in pairs of two young parents or two old parents, implying that these age combinations might prejudice offspring quality. Analysis of the viability of 3,361 offspring of parents up to 21 years old revealed that fledglings with two young parents or two old parents were least likely to become breeders, whereas fledglings with one young parent and one old parent were most likely to do so. For young parents of either sex, offspring viability increased with age of the other parent; for very old parents, it decreased. These effects could be mediated by parents flexibly modifying their investment in offspring in response to their own and their partners´ ages, but evidence for this was lacking. In 5,343 breeding attempts, although mothers’ and fathers’ ages independently affected four heavily care-dependent breeding traits at the clutch and nestling stages, their interaction did not affect any trait. The effects of parental age combinations on viability could also be mediated by genes: fledglings with one young parent and one old parent could benefit from greater heterozygosity or better genes.

## Introduction

It is now well documented that individual animals’ reproductive performance and breeding success commonly improve in early life and decline in late life [1–3]. We have less of an idea about how the viability of an animal’s progeny varies over its lifespan, even though this issue is fundamental for understanding mate choice and modeling life histories. The last 15 years have been characterized by increasing consensus around each of two contrary but non-exclusive theoretical predictions for genetically mediated effects of breeder age on offspring viability [4–6]. On the one hand, differential survival of high quality males and females in wild populations is widely expected to generate a positive association between breeder age and the quality of genes passed to progeny [7–9], although there are also life history arguments for expecting this association to be negative [10,11]. On the other hand, decline over the lifetime is expected in sperm traits of males and in the germline DNA of both sexes, especially males; and these declines should lead to deficits, defects and diseases in offspring [12,13]. Studies have confirmed deterioration in sperm traits [12] and in germline DNA of males and females [14–17]; how these changes affect fertility and offspring viability is largely unresolved [6] but one study has shown that deterioration in male germline DNA after maturity is associated with reduced growth rates in offspring [18]. Furthermore, the viability of offspring can potentially vary with age-related variation in parental investment and capacity for parental care. For example, parental care sometimes improves with age and experience in early life and declines with age and senescence in later life [19–21], raising the possibility of reduced viability in offspring nurtured by very young or very old parents.

Laboratory and field studies focused on either the mother´s age or the father´s age have turned up a mixed bag of positive and negative effects on offspring quality. Advanced male age, even in species with no parental care, is associated with reduced hatching success [11] and poor larval and infant body mass and survival [22,23], as well as diminished activity and learning during infancy and adulthood [24]. However, in some cases superior phenotypes have been observed in the progeny of males that are older [25,26] or middle-aged [27–29]. In invertebrates greater female age is associated with a decline in numerous preadult and adult traits of progeny ([30,31] but see [32]). In birds it is associated with a decline in egg size and in the growth, development and immunity of chicks [21,33], and with accelerated offspring reproductive senescence [34]. In mammals it is associated with negative effects on juvenile survival and daughters’ milk yield [35,36]. However, improvement with age has been documented early in females’ lives too: older mothers produced larger hatchlings in an arachnid [37], higher quality or larger eggs in birds [21,38,39] and heavier offspring with faster reproductive development in mammals [40,41]; and offspring of middle-aged lizards were most likely to survive the juvenile period [28].

Recognition of parental age effects on offspring quality may have been hampered by two nearly universal shortcomings of this literature. First, as noted by other authors [10,11], analyses of offspring quality have largely failed to sample comprehensively across the natural age-span, resulting in frequent confusion between middle age and old age. Often just two age categories are used (young and old) and their relationship to natural age-spans is seldom clear. Second, possible interactions between male age and female age are virtually unexplored. In humans such interactions can affect the probability of birth defects [42,43] and the genetic quality of progeny [44]. In non-human animals, even long-term field studies are generally unable to test for interactions because of the difficulty of obtaining adequate samples of pairs of known-age parents encompassing the age-span, and because partners’ ages are often correlated. In view of recently documented widespread effects of senescence on diverse reproductive traits of wild animals of both sexes [45,34], inclusion of whole natural age-spans and male-female age interactions is required to document age effects more comprehensively.

We analysed effects of male and female ages and interactions between them across their age-spans on offspring viability in the blue-footed booby (*Sula nebouxii*), a socially monogamous species with roughly symmetrical biparental care. The pattern of age-related sexual infidelity observed in these boobies was interpreted as possibly enabling young females to avoid producing offspring that have two young biological parents, and old females to avoid producing offspring that have two old biological parents, because these two parental age combinations might generate poor quality offspring [46]. Extra-pair chicks occur in 11% of broods and the tendency to produce extra-pair chicks increases in very young females (≤4 years old) with decreasing age of social partners, and in old females (≥8 years old) with increasing age of social partners. It is likely that female boobies exert considerable control over extra-pair paternity because they are larger than males and control sexual access, and because extra-pair copulations follow reciprocal courtship and are consensual [47]. Also, female birds can control fertilization through postcopulatory mechanisms [48]. Effects of combined parental ages on offspring have not been explored in this species, but reproductive traits of long-lived male and female boobies improve in early life and show a senescent decline in later life. Their breeding success peaks at ~10 years [49–51] and the viability of their fledglings (probability they will recruit) plateaus when parents are 5–12 years old [52]. Documented traits that may partially explain these patterns include an age-related decline in the quality of eggs and parental care of females [21] and in the germ-line DNA of males [17].

Our test used a 25-year reproductive database for the insular booby population in which parental ages interact to affect extra-pair paternity [46]. In this population annual survival of male and female adults is roughly 80% [53] and average pair bond duration is only 1.7 years [54,55], allowing pairing of different-aged males and females. Adults experience minimal extrinsic mortality (negligible predation [56]) and, for recent fledgling cohorts, most parents’ exact ages are known [57]. Considering the age-related patterns of extra-pair paternity in this population, we predicted that the probability of fledglings recruiting into the breeding population should increase with age of the father for young females but decrease for old females. After confirming this pattern, we tested whether it is due to age combination-related variation in parental investment by asking whether combined parental ages similarly affected any of six breeding traits that are heavily dependent on bi-parental care: clutch size, hatching success, brood size, number of fledglings, fledging success and fledgling body condition.

## Methods

### Ethics Statement

This observational field study was carried out following the Animal Behavior Society's Guidelines for the Use of Animals in Research and in accordance with all Mexican legal requirements for research in national parks and animal conservation and welfare. The study species is not classified as endangered or protected by norm 059–2010 of the Secretaría del Medioambiente y Recursos Naturales (SEMARNAT). The methods of monitoring of boobies in Parque Nacional Isla Isabel were approved by the Dirección General de Vida Silvestre, Secretaría de Gestión para la Protección Ambiental (SEMARNAT permits 517, 574, 5664, 10470, SGPA/DGVS/01323, SGPA/DGVS/3152, SGPA/DGVS/1543, SGPA/DGVS/0491, SGPA/DGVS/1547, SGPA/DGVS/10832, SGPA/DGVS/01916,SGPA/DGVS/00733, SGPA/DGVS/00357, SGPA/DGVS/00505, SGPA/DGVS/00091). During monitoring, adults were not captured or manipulated; chicks were captured by hand after hatching and at fledging for banding and measuring, which was carried out in the shade and took less than 4 min, then immediately returned to their nests, where parents always immediately accepted them.

### Recruitment

We monitored reproduction in a study area on Isla Isabel, off the Pacific coast of Mexico (21°52'N, 105°54'W), during 5 months of each year between 1988 and 2012. Breeding of all pairs of boobies in the study area was monitored by recording nest contents every 3–6 days, banding chicks with plastic bands at hatching and steel bands at fledging (age 70 days), reading bands of caretaking adults at each nest three times (without capturing them), and measuring body mass and ulna length at fledging (details in [57]). Sex of fledglings was unknown. We are confident that this monitoring allowed detection of nearly all recruitment (first appearance with a partner and clutch) of the focal fledglings through 2012. First, over a 19-year period when 10,839 fledglings and 3,160 adults were banded, 40% of fledglings recruited into the study area; and an intermittent study of booby populations in the Islas Marietas, Isla San Pedro Martir and El Rancho island (130–903 km distant) provided resightings of only seven male and six female fledglings breeding elsewhere than Isla Isabel [54]. Further, boobies that recruit into the natal colony breed close to their natal site and then remain permanently faithful to their first breeding site [54]. Second, unless they are abandoned, nests in the study area and within 20 m of its borders cannot escape detection because every year two observers inspected the whole of this greater area every 3–6 days, and in only one direction (South) is there any scope for boobies to nest beyond its limits (and only within a 10-m corridor along the shoreline).

For the first sample comprising seven cohorts (2002–2009 except 2003, from which no fledglings recruited), we scored recruitment (1–0 variable) during the first six years of life up to 2012, and included the number of years during which each cohort was monitored (3–6 years) as a covariate. More than 95% of fledglings recruit by an age of 6 years [57]. We complemented this analysis with an analysis of early recruitment (during the first 4 years of life) in which cohorts 2002–2008 (except 2003) were all monitored for the same number of years. The first sample included 3,361 fledglings with mothers 2–20 years old (mean±SD = 8.28±3.19 years) and fathers 1–21 years old (8.59±3.11 years); the second included 2,819 fledglings with mothers 2–20 years old (8.10±3.11 years) and fathers 1–20 years old (8.32±2.92 years). The first sample was based on 915 individual mothers and 945 individual fathers; the second on 904 individual mothers and 872 individual fathers. Parentage was known from routine monitoring of fledglings´ natal nests and associated caretaking adults.

### Breeding traits

Males and females share all aspects of parental care, including territory defence, incubation and brood care. For analysing the effects of fathers’ and mothers’ ages on clutch size, brood size, number of fledglings, hatching success, fledging success and fledgling body condition, the monitoring described above yielded a sample of 5,343 breeding events of known-age pairs in the study area in the 11 years from 2002 to 2012. Mothers and fathers in this sample were 1–23 years old (8.05±3.87 years and 8.39±3.72 years, respectively).

### Statistical analyses

We analysed effects of fathers’ and mothers’ ages on recruitment and breeding traits using generalized linear mixed models (GLMMs) including fathers’ and mothers’ ages, their quadratic terms and the interaction between the mother’s and father’s linear ages; fledglings’ hatch dates; and hatch order in the natal brood of 1–4 chicks. Mothers’ and fathers’ recruitment ages were not included because these are not related to fledgling recruitment [52], and their lifespans were not included because for most young parents these were not known. To implement a maximal model and reduce Type 1 error, we fitted the intercepts and slopes of the three random variables: mother’s ID, father’s ID and cohort [58]. However, maximal models failed to converge, even when the predictor variables were centered. This failure could not be satisfactorily resolved by deleting males and females that were observed at only a single age because these represented roughly 40% of individuals. Therefore for each model we removed, successively, the slopes for cohort, male ID and female ID until convergence was achieved. For analyses of recruitment all resulting random structures included only the intercepts of random variables; for breeding traits all resulting structures included the intercepts for cohort, male ID and female ID and also the slope for female ID. For hatching success and fledging success, the additional random variable, nest ID, was represented only by its intercept.

To analyse the recruitment of fledglings, we used GLMMs with binomial error distribution and a logit link, including, for the first sample of breeding pairs only, number of years of monitoring as an additional covariate. Ages of male and female partners in the first and the second samples were weakly correlated (R = 0.34, *P* = 0.01, *n* = 3,361; R = 0.31, *P* = 0.01, n = 2,819; respectively), but the Variance Inflation Factor showed no collinearity (GVIF: females,1.12; males,1.13; GVIF: females,1.08; males,1.07; respectively).

To analyse clutch size, brood size and number of fledglings (count data), we used GLMMs with negative binomial error distributions to account for overdispersion [59]. For hatching success and fledging success we used GLMMs with binomial error distribution and a logit link, in which each egg (or chick) was scored as hatching (or fledging) or not and nest ID was an additional random term. For fledgling body condition index, we used a GLMM with normal error distribution and an identity link function; normality was confirmed by graphical analysis of residuals (normal Q-Q plots [60]). For the index we used the residuals of the regression of log body mass on log ulna length at 70 d (linear regression: *F*_1,4871_ = 1946.23, *P*<0.0001, *R* ^2^ = 0.28, *P*<0.001) [61].

In each case, the full model was compared with a model lacking the fixed terms of interest (Mother´s age, Father´s age, Mother´s age^2^, Father´s age^2^, Mother´s age*Father´s age) using a Likelihood Ratio Test. If significant (all were, except the models for clutch size and fledgling body condition), we obtained a final model by deleting the non-significant quadratic and interaction terms. We used version 3.1.0 of R [62] with the glmmADMB library for count data [63] and 3.1.5 of R with the lme4 library for proportions and for index of body condition [64].

## Results

### Recruitment

**Fig 1:**
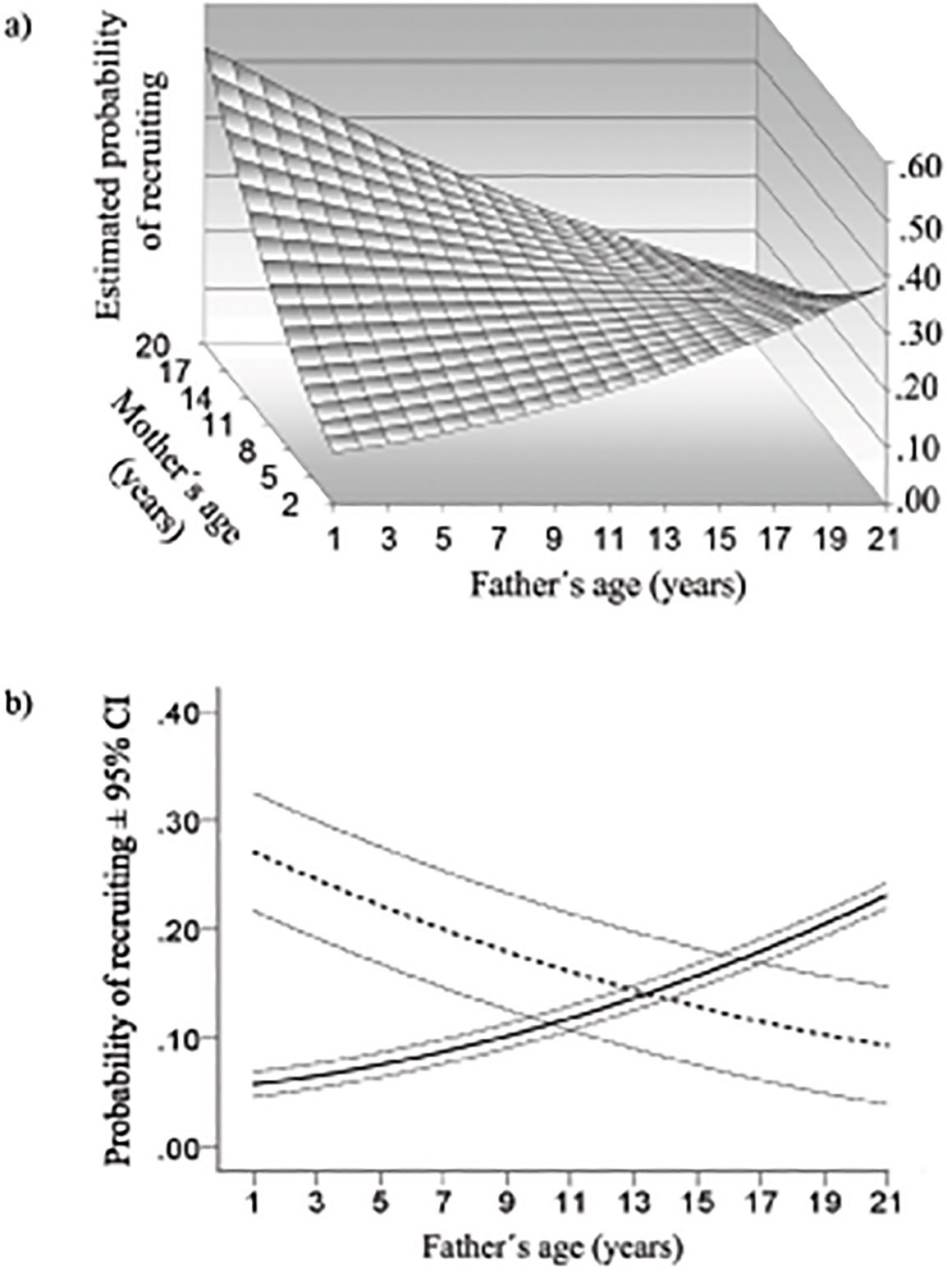
Effect of combined parental ages on estimated probability of recruiting into the breeding population. (a) The surface, generated from a GLMM, is based on 3,361 fledglings (*P* = 0.015). (b) For illustration, predictive curves were generated by GLMM estimates for very young mothers (≤4 years; solid line) and very old mothers (≥13 years; dashed line).

For the first sample (scored over 6 years), the probability of recruitment of fledglings was affected by the interaction of the mother’s and father’s linear ages (*X* ^*2*^_*1*_ = 5.96, *P* = 0.015; Table 1). The interaction was due to offspring with two young parents or two old parents being least likely to recruit, while those with one young parent plus one old parent were most likely to recruit (Fig 1A). Offspring with a young or old parent of either sex were more likely to recruit if their other parent was of the opposite age extreme. For offspring of very young mothers (≤4 years), probability of recruitment roughly doubled when their fathers were very old (≥13 years) rather than very young, whereas for offspring of very old mothers it increased by more than half when their fathers were very young rather than very old (Fig 1B). Recruitment prospects of the offspring of young versus very old fathers were affected by partner age in a similarly contrasting pattern.

**Table 1:** Final models for effects of parents' ages and interactions on the probability of recruitment of fledglings. *P*-values for terms comprised in significant interactions are not shown because they are meaningless. For random factors we show variances (and SD) of intercepts.

Fixed effects	Estimate (SE)	X^2^	*P*
**First sample (scored over 6 years)**			
Intercept	-2.7778 (1.7936)		
Mother´s age	0.1403 (0.0417)		
Father´s age	0.1145 (0.0405)		
Laying date	-1.4780 (0.1914)	59.07	<0.001
Status		17.20	<0.001
(a) Second chick	-0.3465 (0.0927)		
(b) Third chick	-0.4894 (0.1918)		
Years of monitoring	0.1226(0.3326)	0.14	0.714
Mother´s age * Father´s age	-0.0108 (0.0044)	5.97	0.015
σ^2^_father ID_ = 0.0610 (0.2470); σ^2^_mother ID_ = 0.0740 (0.2720); σ^2^_cohort_ = 0.9480(0.9736); σ^2^_residual_ = 1			
**Second sample (scored over 4 years)**			
Intercept	-2.1032 (0.5582)		
Mother´s age	0.1399 (0.0436)		
Father´s age	0.1065 (0.0423)		
Laying date	-1.4817 (0.1971)	55.64	<0.001
Status		16.47	<0.001
(a) Second chick	-0.3489 (0.0956)		
(b) Third chick	-0.4727 (0.1941)		
Mother´s age * Father´s age	-0.0102 (0.0047)	4.68	0.030
σ^2^_father ID_ = 0.0047 (0.0686); σ^2^_mother ID_ = 0.1500 (0.3873); σ^2^_cohort_ = 1.0860 (1.0400); σ^2^_residual_ = 1			

Neither the significant interaction nor the form of the surface in Fig 1 was due to small unrepresentative samples for extreme parental ages: significance of the interaction of parental linear ages was little affected by deleting the offspring of the 7 very old mothers paired to very young fathers (β = -0.0125, SE = 0.0045, *X* ^*2*^_*1*_ = 7.79, *P* = 0.005) or the offspring of the 23 young mothers paired to very old fathers (β = -0.0107, SE = 0.0047, *X* ^*2*^_*1*_ = 5.23, *P* = 0.022), the two parental age combinations least represented in the sample. Sample sizes for parental age combinations are shown in S1 Table.

Results for the second sample (scored over 4 years) were similar in every respect. Probability of early recruitment was affected by the interaction of the mother’s and the father’s linear ages (*X* ^*2*^_*1*_ = 4.68, *P* = 0.030, Table 1), and this effect remained significant after deleting the offspring of very old mothers paired to very young fathers (*n* = 7) or very young mothers paired to very old fathers (*n* = 22) (respectively: β = -0.0102, SE = 0.0050, *X* ^*2*^_*1*_ = 4.682, *P* = 0.031; β = -0.0102, SE = 0.0050, *X* ^*2*^_*1*_ = 4.07, *P* = 0.043). Sample sizes for parental age combinations are shown in S1 Table.

### Breeding traits

**Fig 2:**
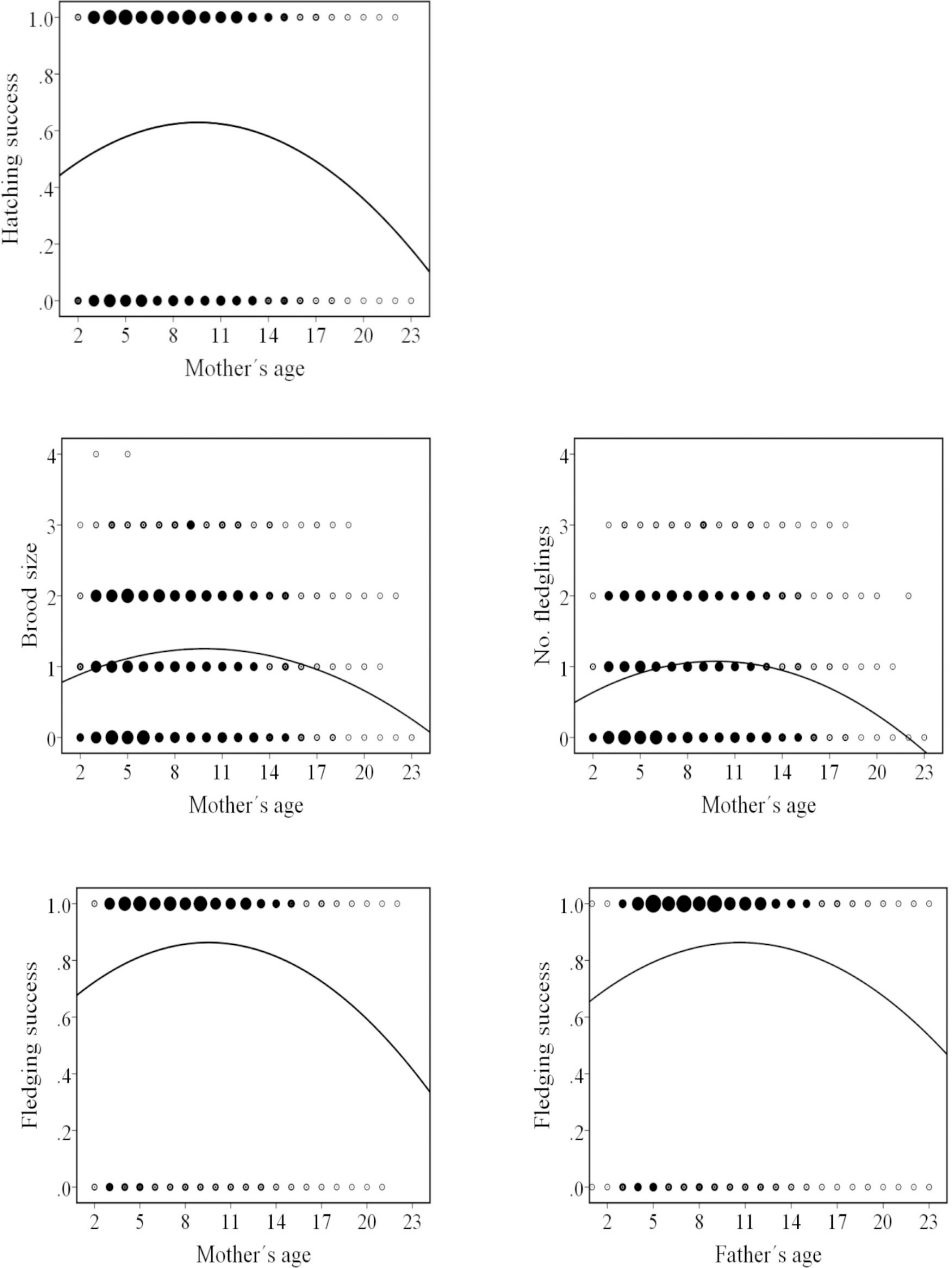
Significant quadratic effects of mothers’ and fathers’ ages (years) on breeding traits.

None of the six breeding traits was affected by the interaction of parents’ linear ages (Table 2) although positive and quadratic effects of either male age or female age were common: hatching success, brood size and number of fledglings were affected by linear and quadratic terms for female age, and fledging success was affected by the linear and quadratic terms for both female and male age (Table 2, Fig 2).

**Table 2:** Final models for effects of parents' ages and interactions between them on breeding traits. *P*-values for terms comprised in significant interactions are not shown because they are meaningless. For random factors we show variances (and SD) for slopes (S) and intercepts (I). Piecewise regression on significant quadratic effects showed that all initial increases and final decreases with age were significant (P<0.05). Effects of ID may have been nonsignificant because individuals were observed with few repetitions; e.g., 2.87±2.37 repetitions for the 1,860 fathers and 3.00±2.58 repetitions for the 1,789 mothers analysed for fixed effects of models 3 and 4.

Fixed effects	*n*	Estimate (SE)	Deviance	*X* ^*2*^	*P*
**1. Clutch size**	5,343				ns
**2. Hatching success**	10,428				
Intercept		1.6884 (0.4798)			
Father´s age		-0.0080 (0.0095)		0.63	0.426
Mother´s age		0.2049 (0.0375)		27.42	<0.001
Mother´s age^2^		-0.0106 (0.0020)		25.38	<0.001
Laying date		-4.9944 (0.1404)		1481.70	<0.001
σ^2^_father ID,I_ = 0.1165 (0.3413); σ^2^_mother ID,I (mother´s age)_ = 0.5820 (0.7630); σ^2^_mother ID,S (mother´s age)_ = 0.0052 (0.0722);					
σ^2^_mother ID,I (father´s age)_ = 0.3584 (0.5987); σ^2^_mother ID,S (father´s age)_ = 0.0024 (0.0488);					
σ^2^_cohort,I_ = 2.1928 (1.4810); σ2_nest ID,I_ = 0.876 (0.9361); σ^2^_residual_ = 1					
**3. Brood size**	5,343				
Intercept		0.0732 (0.1984)			
Father´s age		-0.0014 (0.0378)	0.12		0.729
Mother´s age		0.0740 (0.0156)	26.16		<0.001
Mother´s age^2^		-0.0035 (0.0008)	18.36		<0.001
Laying date		-1.1813 (0.0510)	555.82		<0.001
σ^2^_father ID,I_ = 2.069e-09 (4.549e-05); σ^2^_mother ID,I (mother´s age)_ = 2.065e-09 (4.544e-05);					
σ^2^_mother ID,S (mother´s age)_ = 2.061e-09 (4.54e-05); σ^2^_mother ID,I (father´s age)_ = 2.065e-09 (4.544e-05);					
σ^2^_mother ID,S (father´s age)_ = 2.061e-09 (5.540-e-05); σ^2^_cohort,I_ = 0.3725 (0.6104)					
**4. No. fledglings**	5,343				
Intercept		-0.3035 (0.2627)			
Father´s age		0.0010 (0.0042)	0.66		0.817
Mother´s age		0.1131 (0.0179)	46.62		<0.001
Mother´s age^2^		-0.0054 (0.0010)	34.26		<0.001
Laying date		-1.4539 (0.0567)	686.06		<0.001
σ^2^_father ID,I_ = 2.157e-09 (4.644e-05); σ^2^_mother ID,I (mother´s age)_ = 2.109e-09 (4.592e-05);					
σ^2^_mother ID,S (mother´s age)_ = 2.061e-09 (4.540e-05); σ^2^_mother ID,I (father´s age)_ = 2.209e-09 (4.70e-05);					
σ^2^_mother ID,S (father´s age)_ = 2.209e-09 (4.70e-05); σ^2^_cohort,I_ = 0.6799 (0.8245)					
**5. Fledging succes*s***	6,125				
Intercept		1.2456 (0.4610)			
Father´s age		0.1651 (0.0495)		8.89	0.011
Father´s age^2^		-0.0077 (0.0024)		8.78	0.003
Mother´s age		0.1524 (0.0528)		6.19	0.045
Mother´s age^2^		-0.0080 (0.0030)		6.01	0.014
Laying date		-2.9791 (0.1828)		267.97	<0.001
σ^2^_father ID,I_ = 0.0818 (0.2860); σ^2^_mother ID,I (mother´s age)_ = 0.8284 (0.9101); σ^2^_mother ID,S (mother´s age)_ = 0.0104 (0.1020);					
σ^2^_mother ID,I (father´s age)_ = 0.0000 (0.0000); σ^2^_mother ID,S (father´s age)_ = 0.0011 (0.0327);					
σ^2^_cohort,I_ = 1.4612 (1.2198); σ2_nest ID,I_ = 0.3086 (0.5555); σ^2^_residual_ = 1					
**6. Fledgling body condition **	4,873				ns

## Discussion

In the blue-footed booby, offspring viability, measured as probability of recruitment or early recruitment into the breeding population, depends strongly on the combination of parental ages. Fledglings with two very young parents (≤4 years) or two very old parents (≥13 years) were the most likely to fail, whereas fledglings with one very young parent and one very old parent were the most likely to succeed. For a young parent of either sex offspring viability increased with age of the partner, while for a very old parent of either sex offspring viability decreased with age of the partner. Failure to recruit could occur because fledglings died before the age of recruitment or because they were insufficiently competitive adults to acquire a territory and partner and care for a clutch. The phenotypic traits of fledglings associated with this pattern were not identified; they may not include body condition at fledging because this variable was not affected by a parental age interaction. Effects of combined parental ages across the age-span on offspring quality have not, to our knowledge, been investigated in other non-human species except in a study of semi-captive common lizards (*Lacerta vivipara*), where parental ages did not interact to affect first-year survival [28].

Variation in viability of fledglings could be due to variation in parental investment. At the oocyte/egg stage, female birds can influence diverse aspects of offspring development both epigenetically and by modifying hormone, carotenoid and antibody contents of yolk [65–67], and both sexes can also influence development by adjusting their care of clutch and brood. Furthermore, in some circumstances female and male parents are expected to modify their investment in progeny in response to the characteristics of their mates [68,69], including age [70]. According to one model, adjustments made by female animals in response to male quality can depend on the female’s own age or body condition, for example with young females increasing investment when the male is of high quality and old females doing so when he is of low quality [71]. Consistent with this expectation, young female mallards (*Anas platyrhynchos*) laid larger eggs when their partner was attractive [72] whereas old female mallards in another study laid larger eggs when their partner was unattractive [73]. In theory then, the high viability of fledgling boobies with dissimilar-aged parents could be explained by young mothers increasing investment in eggs or care of clutch and brood when paired with old males, and old mothers doing so when paired with young males; or similar adjustments by fathers.

However, scrutiny of thousands of breeding attempts failed to provide any evidence that parental ages interact to affect parental investment. Four of six breeding traits measured during the period of parental care showed highly significant quadratic and linear effects of female age, and fledging success also showed highly significant quadratic and linear effects of male age, but none of the six was affected by the interaction of male and female ages (Fig 2, Table 2).Thus the pattern of parental age effects at the egg and nestling stages did not anticipate the pattern of their effects on fledgling recruitment. This implies that effects of parental age combination on recruitment may not be mediated by their effects on parental investment. Further, although age-sensitive adjustments in investment can potentially explain pairs of young or old breeders producing low-viability fledglings, they cannot explain them producing a high proportion of extra-pair chicks [46]. The latter finding calls for an explanation by a genetic mechanism because extra-pair fathers contribute no parental investment to offspring, only genes.

Identification of the benefits of extra-pair behaviour to female birds has been troublesome [74], but there are good grounds for believing that they sometimes accrue through pairing females’ genes with male genes that complement them better than those of their social partners [48]. It is possible that very old female boobies most frequently produce extra-pair chicks when paired to very old males, and young females when paired to young males, in order to avoid saddling their progeny with the gene combinations brought about by those age pairings [46]. If we assume that a genetic mechanism underlies the pattern of parental age-related fledgling viability, then by obtaining extra-pair sires for their offspring that are an average of 6.3 years older than their social partners [46], young females could increase their fledglings’ chances of recruiting by roughly 50% (estimated from Fig 1B).

Interacting effects of parental ages on offspring quality have been reported in humans, and genetic or gene-environment causes are suspected. Offspring of old fathers are more likely to have diseases of genetic origin [75], and the probability of birth defects of known or suspected genetic origin varies in complex ways with the combination of maternal and paternal ages [42,44]. We speculate that the high viability of fledglings with dissimilar-aged parents could derive from increased heterozygosity or high quality genes. In populations with overlapping generations, great environmental fluctuations such as those of El Niño Southern Oscillation can maintain variation in genes, including genes of large effect [76,77], and nestlings whose parents belong to temporally distant birth cohorts exposed to different selection regimes over different periods of time may be more heterozygous. Alternatively, high genetic quality could be favored in offspring of dissimilar-aged parents by the two age-related mechanisms most widely expected to affect viability of animal offspring: increase in the average genetic quality of surviving breeders [7–9] and decrease in the integrity of adult germ line DNA [12,13]. Assuming damage to DNA is greater in the germ line of males than females [78,79], and that the capacity of oocytes to repair DNA damage in male sperm declines with female age [13,80], superior viability could be shown by (1) offspring of young female/old male matings because of old males’ high genetic quality coupled with young females’ ability to repair their damaged DNA, and (2) offspring of old female/young male matings because of old females’ high genetic quality and relatively undamaged DNA. Fledglings derived from young/old parent matings could be the most viable because they are the only ones to receive high quality genes with relatively undamaged or substantially repaired DNA from one parent along with undamaged DNA from the other parent.

If the observed pattern of variation in offspring viability is due to genetic effects, then our analyses may well underestimate the impact of parental age combinations on offspring viability, because our sample of fledglings surely included a proportion of individuals sired by females’ extra-pair partners. Based on rates of extra-pair paternity observed in this population [46], an estimated 20.3% of fledglings with two very young parents and 20.8% of fledglings with an old mother (≥8 years) and a very old father were probably sired by males of less extreme age than the social father. Such extra-pair fledglings should inflate the model’s estimates for recruitment of offspring from those two parental age combinations because they derive from more favorable combinations. A quite different interpretation is that the low average viability of offspring of two old parents or two young parents is due to the relatively high incidence of extra-pair individuals, if we assume that extra-pair individuals have low viability. However, it is implausible that female boobies would allow, let alone foster, extra-pair fertilizations that greatly reduce the viability of their progeny.

We should seek parental age combination effects in other species and explore their consequences for mating systems. Although they can be large (Fig 1), combination effects may have gone undetected because of the difficulty of obtaining adequate samples, particularly of old individuals paired with variably aged partners. Wherever there is an effect of parental age combination on progeny, whether due to variation in parental investment or in offspring genomes, females’ and males’ own ages are likely to affect their preferences among breeding partners of different ages. Where combination effects are due to offspring genomes, the particular age combination of a breeding pair could potentially affect both partners’ extra-pair behaviour and their willingness to invest in their shared offspring. For example, suboptimal age combinations could favor females seeking extra-pair sires of ages that combine better with their own ages (cf. [46]), and males increasing their relative investment in extra-pair versus within-pair reproduction.

## Supporting Information

S1 Table Sample sizes for analysis of recruitment of fledglings with different combinations of mothers’ and fathers’ ages. (XLS)
